# Feeding global aquaculture

**DOI:** 10.1126/sciadv.adn9698

**Published:** 2024-10-16

**Authors:** Spencer Roberts, Jennifer Jacquet, Patricia Majluf, Matthew N. Hayek

**Affiliations:** ^1^ Department of Environmental Science and Policy and Abess Center for Ecosystem Science and Policy, University of Miami, Miami, FL, USA.; ^2^ Science and Strategy Team, Oceana, Washington, DC, USA.; ^3^ Science and Strategy Team, Oceana, Lima, Peru.; ^4^ Department of Environmental Studies, New York University, New York, NY, USA.

## Abstract

The growth of animal aquaculture requires ever more feed. Yet, fish and crustacean farming is argued to be sustainable because wild fish use is low and has improved over time. Here, accounting for trimmings and by-products from wild fish in aquaculture feed, and using four different sources of industry-reported feed composition data, we find ratios of fish inputs to farmed outputs of 0.36 to 1.15—27 to 307% higher than a previous estimate of 0.28. Furthermore, a metric that incorporates wild fish mortality during capture and excludes unfed systems raises the wild fish mortality–to–farmed fish output ratio to 0.57 to 1.78. We also evaluate terrestrial ingredients in aquaculture feeds. Widely cited estimates of declines in wild fish use from 1997 to 2017 entailed a trade-off of more than fivefold increase in feed crops over the same period. Our assessment challenges the sustainability of fed aquaculture and its role in food security.

## Teaser

Global fish farming relies on wild-caught ocean fish, in quantities larger than previously reported.

## INTRODUCTION

The farming of many aquatic species relies on feed, which incurs social and environmental impacts. An issue that has raised much attention is the reliance on capture fisheries for reduction into fish meal and oil. “Reduction fisheries”—industrial seine fleets targeting small pelagic fish such as anchoveta, whiting, and sardine—account for an estimated average of one-sixth of the mass of the global marine catch and can comprise nearly one-third in some years (*1*–*3*). Approximately 70% of this biomass is processed into aquaculture feed, with the remaining 30% being used for other animal feed, supplements, and cosmetics (*4*). Reduction fisheries have global ecological impacts (*5*), affecting the structure of exploited populations (*6*) and reducing food availability for predators, such as fish, seabirds, and marine mammals (*7*). Reduction fishing also diverts millions of tons of food-grade fish (e.g., anchovies and sardines) and nutrients from countries with high rates of hunger to farmed aquatic animals (e.g., salmon and shrimp) intended for luxury markets (*8*–*11*).

The fish-in:fish-out (FI:FO) metric was developed to quantify the reliance of aquaculture on captured fish. FI:FO is distinct from the feed conversion ratio (FCR) metric, which divides dehydrated feed inputs by wet fish outputs. FI:FO reconstructs a “live weight equivalent” from reported feed use, which approximates the biomass of wild fish consumed, then divides it by farmed output to estimate the ratio of fished biomass inputs to farmed fish biomass outputs for a given farm, farmed species group, or the aquaculture sector as a whole (*12*). FI:FO quantities should therefore reflect integrated average wild fish utilization across all stages of the farmed fishes’ life cycles.

The FI:FO metric has been through several iterations. Revisions have been made to account for “residual” and “recovered” oil embedded in fish meal or coming from the same fish that were made into fish meal, though debates persist surrounding how to apportion these subtractions between divisions of the aquaculture industry farming different animal taxa (henceforth: “species groups”). A method emerged, established by Hardy in 2009 (*13*), which was used by Naylor *et al.* (*14*) in 2021 to analyze Food and Agriculture Organization of the United Nations (FAO) reports and industry surveys and compare FI:FO ratios from 1997 and 2017 across the top 11 feed-consuming species groups. These methods yielded results that showed that the intensity of wild fish use in fish and crustacean farming fell by 85% over 20 years.

However, translating processed feed inputs into a live weight equivalent of wild fish requires making approximations for proportions of wild fish incorporated in aquaculture feeds and assumptions regarding how they are reduced and processed. Naylor *et al.* (*14*) compiled a comprehensive estimation of these feed reduction parameters, concluding that aquaculture feed, on aggregate, consisted of approximately 7% wild fish in 2017. Yet, the proprietary status of feed manufacturing requires taking these data from voluntary industry disclosures, which are difficult to validate. We compiled additional feed composition datasets obtained using survey, projection, or metastudy for similar time frames by the FAO (*15*), Monterey Bay Aquarium (MBA) Seafood Watch (*16*–*25*), and Pahlow *et al.* (*26*). A wider range of source-independent estimates may help gauge uncertainty, as well as risk.

With respect to methods for calculating FI:FO, common conventions in scholarly literature omit two substantial sources of wild fish. First, recovered oil (oil produced during fish meal processing) is typically calculated assuming no losses and subtracted entirely (*13*, *14*), but industry reports indicate that an estimated 27% of this oil is not used by aquaculture, but instead allocated to human supplements, terrestrial animal feeds, cosmetics, and other uses (*27*). Second, all meal and oil from trimmings—parts of marine animals’ bodies that are removed during processing—are conventionally classified as by-products and therefore subtracted on the basis that they are not wild fish inputs. Furthermore, whole fish are increasingly classified as “trash,” particularly when caught while targeting other species, and reported entirely as by-product (*28*, *29*). However, industry reports estimate that roughly two-thirds of trimmings and by-products (henceforth: “trimmings”) are sourced from wild-captured fish (*27*).

Beyond inputs, reduction fisheries incur high rates of fishing mortality due to “slipping” (*30*–*36*)—when vessels leave purse seine nets slightly open to allow unwanted catch to escape, which results in estimated postrelease fish mortality rates ranging from 38% to more than 99% in trials (*36*–*42*). The collateral mortality involved in slipping, along with additional “bycatch” from fisheries from which trimmings are sourced, has not been considered in FI:FO formulations to date. Last, we adjust the formula to approximate the efficiency of the aquacultural feeding process, as opposed to total industrial inputs over outputs, considering that more than a quarter of farmed fish and crustacean biomass is derived from systems that do not use compound feeds (henceforth: “unfed”).

It is also important to note that any metric for assessing the reliance on fish meal and oil is not a comprehensive metric for assessing the sustainability of aquaculture feed because these calculations do not include the reliance of aquaculture on terrestrial feed inputs (*43*). Terrestrial feed production is linked to generation of emissions and effluent, as well as freshwater consumption (*26*), land use changes (*44*), and opportunity costs (*45*). Here, we aim to account for previous omissions and trade-offs to provide a more comprehensive environmental evaluation of the feed requirements for global aquaculture. These methods can be used to inform future research for more comprehensive life cycle assessments.

## METHODS

### Marine-sourced feed

The calculation of *FI*:*FO* can be defined as follows, where *LWE* is the live weight equivalent of fish processed into meal or oil, denoted by the subscripts *FM* or *FO*

$$\mathrm{FI}={\mathrm{LWE}}_{\mathrm{FM}}+{\mathrm{LWE}}_{\mathrm{FO}}$$ (1)

The total wild fish inputs (*FI*) are then divided by reported production (*FO*) to yield a *FI*:*FO* quotient. There are multiple approaches, however, for calculating these variables.

### Replicated literature estimate

We first replicated the methods and results of Naylor *et al.* (*14*), which used 2017 as a study year, assumed no waste of recovered fish oil and treated it all as aquaculture inputs, excluded all inputs from trimmings, and did not include collateral fishing mortality (data S1). The below formula, which disambiguates the variables above to estimate wild fish inputs for a given farmed species group (*i*), is adapted from Naylor *et al.* (*13*), where *FCR* is the feed conversion ratio, *FM_wf_* and *FO_wf_* are the proportions of fish meal/oil derived from whole fish sourced from the wild, *LWE_FM_* is the live weight equivalent for fish meal, and *RE* is the reduction efficiency for fish meal or oil, denoted as abbreviations in subscript. For this calculation, authors use a constant 24% for *RE_FM_* and 5% for *RE_FO_*.

$$\begin{array}{cc} \mathrm{FI} & =\left[{\mathrm{FCR}}_{\left(i\right)}\times \frac{{\mathrm{FM}}_{\mathrm{wf}\left(i\right)}}{{\mathrm{RE}}_{\mathrm{FM}}}\right]+\left[{\mathrm{FCR}}_{\left(i\right)}\times \frac{{\mathrm{FO}}_{\mathrm{wf}\left(i\right)}}{{\mathrm{RE}}_{\mathrm{FO}}}\right]\\ & -\left({\mathrm{RE}}_{\mathrm{FO}}\times {\mathrm{LWE}}_{\mathrm{FM}}\right) \end{array}$$ (2)

### Adjusted estimates

We then developed an adjusted method to quantify wild fish biomass used in feed by incorporating overlooked sources of wild fish inputs. First, we adjusted recovered fish oil so that it did not subtract oil used by other industries, multiplying by 73%—the proportion of fish oil used for aquaculture in 2016 according to the Marine Ingredients Organization (IFFO) (*27*). Recovered oil was also adjusted to multiply the live weight equivalent of fish meal for a given farmed species group by the production efficiency of fish oil (*PE_FO_*)—the ratio of fish oil produced in the global economy relative to the live weight equivalent of produced fish meal (18.1%, as reported by the IFFO)—as opposed to the reduction efficiency—the theoretical maximum quantity of fish oil that could be collected at fish meal processing centers without losses. Then, we included wild-sourced trimmings, multiplying the proportion of trimmings in meal/oil (*FM_t_*/*FO_t_*) by the proportion of wild-sourcing across the industry (*27*), then dividing by the reduction efficiencies for meal/oil sourced from trimmings (*RE_FMt_*/*RE_FOt_*).

$$\begin{array}{cc} \mathrm{FI} & ={\mathrm{FCR}}_{\left(i\right)}\times \left[\frac{{\mathrm{FM}}_{\mathrm{wf}\left(i\right)}}{{\mathrm{RE}}_{\mathrm{FM}}}+\frac{{\mathrm{FM}}_{t\left(i\right)}}{{\mathrm{RE}}_{\mathrm{FMt}}}\right]+{\mathrm{FCR}}_{\left(i\right)}\times \left[\frac{{\mathrm{FO}}_{\mathrm{wf}\left(i\right)}}{{\mathrm{RE}}_{\mathrm{FO}}}+\frac{{\mathrm{FO}}_{t\left(i\right)}}{{\mathrm{RE}}_{\mathrm{FOt}}}\right]\\ & -0.73\times \left({\mathrm{PE}}_{\mathrm{FO}}\times {\mathrm{LWE}}_{\mathrm{FM}}\right) \end{array}$$ (3)

For this equation, we also adjusted reduction efficiency rates by multiplying reported processing yields for each fish taxon (*46*) by the proportions of meal and oil derived from each (*27*), returning a fish meal reduction efficiency for whole fish (*RE_FM_*) of 20.9%, a fish oil reduction efficiency for whole fish (*RE_FO_*) of 7.6%, a fish meal reduction efficiency for trimmings (*RE_FMt_*) of 19.7%, and a fish oil reduction efficiency for trimmings (*RE_FOt_*) of 10.7% (data S1). These reduction efficiency values are used in all subsequent scenarios for likewise comparison, but are not constants and allow the capacity to factor for variations in species composition of fish processed into meal and oil in a given year. For clarity, inputs from reduction fisheries and trimmings are reported respectively as “whole fish” and “fish cuts” in the Results.

This process represents the “adjusted” method. Biomass of wild fish inputs were calculated in two components: whole fish and fish cuts; both separated further into fish meal and fish oil subcomponents. These two components were summed and divided by aggregate weight of farmed animals to yield *FI*:*FO* ratio by farmed species group and across the industry as a whole for each of the five scenarios.

### Biomass of whole fish from the wild

Biomass of whole fish from the wild was calculated using FAO global aquaculture production data for the top 11 feed-intensive aquaculture species groups, from data compiled by Tacon for the year 2017 (*47*), which were multiplied by economic FCRs (feed use divided by production) from the same source. The product of these data estimated total feed use by farmed species group, which were multiplied by the proportions of meal and oil included in feed by farmed species group and again by the proportions of meal and oil from capture fisheries. These proportions of meal and oil from the wild were divided by reduction efficiencies for meal and oil to reconstruct the live weight equivalent of whole fish from the wild. Recovered oil was then taken as an aggregate sum, pooled between all species groups and subtracted from the total. It is not subtracted in calculations for specific species groups, as it is not possible to accurately apportion.

### Biomass of fish cuts from the wild

Next, we calculate biomass of trimmings from the wild, which have previously been entirely subtracted from methods for converting feed usage to wild-caught fish. Instead, we start from total feed use as calculated above, which was multiplied by the proportions of meal and oil included in feed and, this time, by the proportions of meal/oil from trimmings instead of capture fisheries. These figures were multiplied by 65.9%, the proportion of trimmings for meal and oil reported as wild sourced by the IFFO in 2016. These proportions of meal and oil from the wild were divided by 19.7 and 10.7% (*RE_FMt_* and *RE_FOt_*, respectively), as processors report extracting different amounts of product from fish trimmings sold for reduction (data S1).

Therefore, proportions of meal and oil from trimmings (*FM_t_*/*FO_t_*) were calculated for each species group (*i*) by subtracting the proportion of whole fish in feed from one and multiplying by 0.659 the proportion of trimmings and by-products sourced from the wild for the study year

$${\mathrm{FM}}_{t\left(i\right)}=0.659\times \left(1-{\mathrm{FM}}_{\mathrm{wf}}\right)$$ (4)

$${\mathrm{FO}}_{t\left(i\right)}=0.659\times \left(1-{\mathrm{FO}}_{\mathrm{wf}}\right)$$ (5)

### Feed composition scenarios

Each of the five mathematical methods for calculating *FI*:*FO* was iterated using a total of four feed composition scenarios. Data from all sources were chosen as close to the 2017 study year as possible:

1) Naylor&al: We used fish meal and oil inclusion rates as compiled by Naylor *et al.* (*14*), which compile data from the National Research Council in 2011 (*48*) and Ytrestøyl *et al.* (*49*). We then replicated their results via the Hardy *FI*:*FO* method (*13*).

2) Naylor&al*: We used all the same data as Naylor *et al.* (*14*), but applied the adjusted *FI*:*FO* mathematical method. All subsequent scenarios use this method.

3) FAO: We used United Nations FAO projections for fish meal and oil inclusion rates in feed for the year 2015, which were extrapolated from trends in industry surveys published by Tacon *et al.* (*15*) in 2011.

4) MBA: We used MBA Seafood Watch reports for representative species in major producing countries (*16*–*25*), which report both inclusion rates of fish meal/oil and proportions of each sourced from the wild; thus, these were used in calculations for this scenario. These reports are generally more contemporary than those used in other scenarios, published or updated between 2017 and 2023, save for one document from 2014. At the time of publication, carp was being actively evaluated; thus, these values were obtained by direct communication and there was no Seafood Watch assessment for milkfish; hence, to calculate totals in this dataset, conservative figures from the Naylor&al scenario were substituted. The report for bluefin tuna was also included as a case study of an especially fish-intensive species group. This species group is excluded from industry-wide calculations to maintain congruous comparison across scenarios.

5) Pahlow&al: Last, we used fish meal and oil inclusion data compiled using metastudy by Pahlow *et al.* (*26*) in 2015, binning species into their respective species groups and weighting each by their component of reported production (*47*).

### Fish mortality and feed efficiency

#### Adjusted plus collateral

Further, we evaluate biomass of reduction fishing collateral, calculated in two subcomponents: first, by estimating unaccounted mortality from slipping in small pelagic purse seine fisheries. This was calculated in data S1 by compiling slipping rates and postslipping mortality from the literature for species exploited for reduction to fish meal and oil. The mean figures reported by these studies were multiplied to produce a mean slipping mortality rate for each taxon, which was weighted by the respective proportion of each in wild sourcing for fish meal and oil in 2016, as compiled by the industry group SEAFISH from disclosures by Biomar, Skretting, and Cargill (*27*), each weighted equally. This industry-wide mean slipping mortality of 11.1% was added to the mean small pelagic bycatch rate of 1.2% reported by the FAO (*50*)—yielding a total collateral mortality rate of 12.3%—and multiplied by the total biomass of reduction fishery inputs for each farmed species group.

Second, biomass of collateral mortality from trimming fisheries was calculated using a global average 10% bycatch rate (*51*), which is conservative as high-bycatch gear methods such as seafloor trawling comprise the fisheries contributing to trimmings used for fish meal and oil. This rate was multiplied by the total biomass of trimmings from the wild for each farmed species group.

Collateral fishing mortality is represented as the variable *F_B_*, calculated as follows, where *LWE* stands for live weight equivalent and subscripts abbreviate fish meal, fish oil, and trimmings. The bycatch rates used for reduction fisheries and trimmings fisheries are 0.123 and 0.1, respectively

$$\begin{array}{cc} F_{B} & =\left({\mathrm{LWE}}_{\mathrm{FM}}+{\mathrm{LWE}}_{\mathrm{FO}}\right)\times 0.123+[LWE_{T\left(FM\right)}]\\ & +\left[LWE_{T\left(FO\right)}\right]\times 0.1 \end{array}$$ (6)

#### Adjusted minus unfed farms

We adjust the denominator of the ratio to more accurately reflect the efficiency of the fish or crustacean feeding process. Fed fish out, or *FO_f_*, is taken for each species group (*i*) as the total production multiplied by the proportion produced with compound feed (*p_f_*), as compiled by Naylor *et al.* (*14*) in 2021.

$${\mathrm{FO}}_{f}={\mathrm{FO}}_{\left(i\right)}\times p_{f\left(i\right)}$$ (7)

#### Adjusted plus collateral minus unfed farms

Last, we combine all factors to incorporate reduction efficiencies adjusted for species composition of catch, recovered oil adjusted for processing losses, trimmings from wild fish, collateral fishing mortality, and adjustments for fed systems only.

$$\frac{\mathrm{FI}+F_{B}}{{\mathrm{FO}}_{f}}$$ (8)

### Terrestrial-sourced feed

#### Environmental impacts by farmed species group

To quantify terrestrial feed inputs and associated environmental impacts, species-specific aquaculture feed composition data compiled by Pahlow *et al.* (*26*) were weighted by FAO aquaculture species production data for the same year to derive detailed feed composition by specific agricultural commodity for each species, binned to match the species groupings used above for marine feed (*15*). These data were multiplied by land use intensity figures calculated by Poore and Nemecek (*44*) in 2018 and divided by FAO production data used above (*14*) for consistency, yielding land use intensity by species group. Where figures for feed commodities did not exist in the dataset, those for the analogous whole crop were used as approximates. Pahlow *et al.* (*26*) calculations on “blue water” withdrawals were also replicated to allow comparison across species groups. Land and water use for fish processing and aquaculture facilities were not captured within this scope.

#### Crop use over time

To evaluate impacts of a shift from marine to terrestrial-sourced feed over time, biomass of total crop use was estimated for the years 1997 and 2017. To make a congruous comparison, feed composition and conversion data from Naylor *et al.* (*14*) were used for 2017 and data from Naylor *et al.* (*52*) were used for 1997. As feed crop composition data were not available, estimated values for “crop use” encompass small proportions of terrestrial animal products and additives, such as vitamins, minerals, and medications. Proportion of feed composed of fish meal and oil was subtracted from total feed use, calculated by multiplying FAO figures for fed fish production and economic feed conversion rates. Corollary data were not available for intermediate years. The magnitude of these increases in crop use were compared with growth in production for each species group and for the farmed fish and crustacean sector as a whole.

## RESULTS

### Marine-sourced feed

**Fig. 1.**
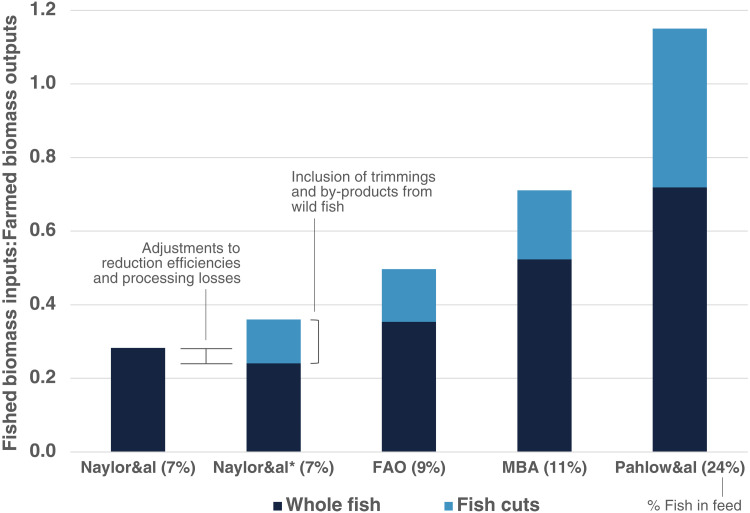
Estimated ratios of wild fish biomass inputs to farmed fish biomass outputs (FI:FO) for global aquaculture across five different datasets and two different methods. Naylor&al uses methods of Naylor *et al.* (*14*). Naylor&al* uses alternative efficiency rates of fish meal and oil processing, assumptions regarding processing waste, and allocations of wild fish cuts. The other datasets differ by meal and oil inclusion rates in feed, and for MBA, the percent of each from by-products. Data were selected as close to 2017 as available. Sources are Naylor *et al.* (*14*), Tacon *et al.* (*15*), Monterey Bay Aquarium Seafood Watch (*16*–*25*), and Pahlow *et al.* (*26*).

Across the top 11 farmed species groups in 2017, the ratio of fish inputs to farmed outputs is at least 27% and up to 307% higher than previously reported, yielding a fished-biomass-in:farmed-biomass-out ratio (FI:FO) of 0.36 to 1.15. Across the feed composition datasets, the Naylor&al scenario demonstrates approximately 7% fish utilization (5.9% meal, 1.3% oil), FAO is 9% (7.2% meal, 1.9% oil), MBA is 11% (8.4% meal, 2.7% oil), and the Pahlow&al scenario is 24% fish (20.5% meal, 3.6% oil) (Fig. 1).

After weighting the yield from processing each taxon of small pelagic fish by its reported proportion in each input stream (data S1), we calculate that the Naylor&al scenario overestimates the efficiency of meal processing, yet underestimate the efficiency of oil processing. This finding substantially reduces the live weight equivalent of consumed feed, yet in aggregate, these deductions (applied in Naylor&al* and subsequent scenarios) are still outweighed by incorporating unaccounted wild fish inputs from trimmings and by-products.

In the Naylor&al* scenario, using the same feed composition data as Naylor *et al.* (*14*), but accounting for recovered fish oil not used for aquaculture and trimmings from wild-caught fish, increased the FI:FO ratio by 29% above that reported by Naylor *et al.* (*14*) (Naylor&al scenario), from 0.28 up to 0.36 for all farmed fish species in aggregate. This is despite our use of an efficiency rate for oil processing in the Naylor&al* scenario of 7.6%—more than 50% higher than used in the Naylor&al scenario (5%), which substantially reduced the live weight equivalent of fish oil (Table 1).

**Table 1.** Estimated ratios of wild fish biomass inputs to farmed fish biomass outputs (FI:FO) for top aquaculture species groups under five different scenarios. The leftmost column represents results of the FI:FO formula outlined by R. W. Hardy and used in Naylor *et al.* (*14*). Fish inputs not captured by this method are included in Naylor&al* and subsequent scenarios. Collateral fishing mortality is not added and unfed systems are not subtracted here. Datasets differ in terms of efficiency rates of fish meal and oil processing, meal and oil inclusion in feed, and, for MBA, percent of each from by-products. Figures are selected as closely to 2017 as available. Sources are Naylor *et al.* (*14*), Tacon *et al.* (*15*), Monterey Bay Aquarium Seafood Watch (*16*–*25*), and Pahlow *et al.* (*26*). Crustaceans and miscellaneous species groups were assessed separately.

Species group	Biomass of wild fish inputs to farmed fish outputs (FI:FO)					Production (kilotons)
	Naylor&al	Naylor&al*	FAO	MBA	Pahlow&al	
Carp	0.02	0.03	0.04	0.07	0.16	13,986
Tilapia	0.03	0.11	0.11	0.21	0.85	5,881
Milkfish	0.07	0.09	0.25	–	0.94	1,729
Catfish	0.02	0.08	0.11	0.43	1.48	5,519
Misc. freshwater fish	0.38	0.49	0.61	1.42	0.73	2,491
Freshwater crustaceans	0.43	0.64	0.64	0.10	1.61	2,536
Shrimp	0.82	0.91	0.93	1.13	1.53	5,512
Misc. marine fish	1.25	1.32	1.90	3.64	3.93	3,098
Trout	1.82	1.51	2.57	1.73	3.69	846
Salmon	1.87	1.66	2.57	3.15	5.57	2,577
Eel	2.98	3.23	3.03	3.85	3.21	259
Bluefin tuna	–	–	–	25.6	–	22.7
Herbivorous†	0.02	0.06	0.06	0.13	0.48	27,115
Carnivorous‡	1.94	2.27	2.60	2.88	4.97	3,682
All	0.28	0.36	0.50	0.71	1.13	44,434

†Herbivorous species groups include carp, tilapia, milkfish, and catfish.

‡Carnivorous species groups include trout, salmon, and eel for the MBA scenario.

Substituting alternative parameters projected by the FAO for 2015, which increased the aggregate amount of fish meal and oil in fish feed by a respective 1.2 and 0.6% above the adjusted Naylor&al* scenario, resulted in a 39% increase in FI:FO to 0.50, demonstrating the sensitivity of this metric to errors, uncertainties, and underreporting. Using the MBA parameters for fish meal/oil inclusion and proportions sourced from the wild, the aggregate FI:FO roughly doubled to 0.71. Using the Pahlow *et al.* (*26*) parameters, which report considerably higher fish meal use, aggregate FI:FO came to 1.15, with total consumed biomass surpassing produced biomass across the industry (Table 1).

The industry-wide FI:FO largely reflects the predominance of herbivorous fish in global aquaculture, particularly carp, which are fed diets consisting of virtually no fish oil, if they are fed at all. For carnivorous species groups (above 5% minimum fish inclusion), such as trout, salmon, and eel, fish biomass inputs exceeded twice the farmed fish biomass produced in most adjusted scenarios. Across our full range of estimates, FI:FO exceeded an aggregate of 2 for carnivorous feed in all scenarios (ranging from 2.27 to 4.97) and 25 for the fish-intensive case study of bluefin tuna (Table 1).

### Fish mortality and feed efficiency

**Fig. 2.**
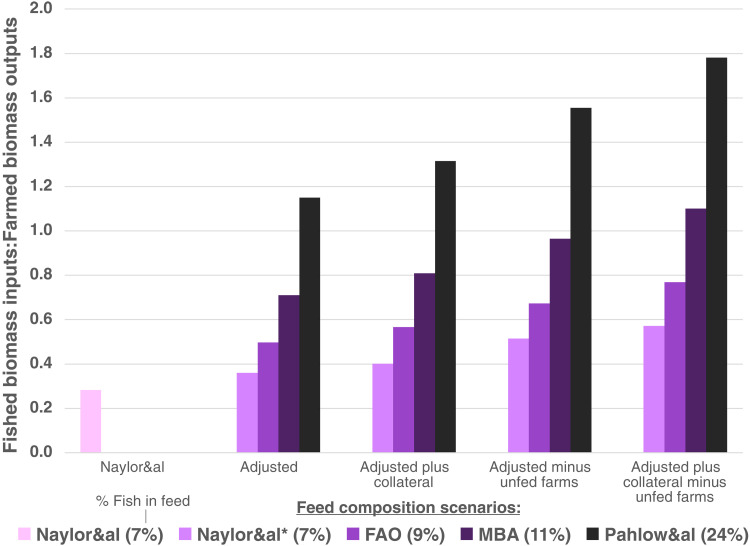
Calculation of wild fish use efficiency in aquaculture by five different mathematical methods using four different sources of feed composition data. Naylor&al shows the results using methods of Naylor *et al.* (*14*). Adjusted methods calculate reduction efficiencies using species composition of reported catch, account for losses of fish oil in processing, and include trimmings and by-products from wild fish. Collateral methods additionally include bycatch. The two rightmost column groupings exclude production from unfed farms. Feed composition data sources are Naylor *et al.* (*14*), Tacon *et al.* (*15*), Monterey Bay Aquarium Seafood Watch (*16*–*25*), and Pahlow *et al.* (*26*).

Incorporating collateral mortality during fish capture increased FI:FO ratios by an additional 12 to 14% across scenarios, yielding a biomass ratio of wild capture mortality to farmed animals ranging from 0.40 to 1.31. Considering only inputs and outputs to fed systems yielded feeding efficiency ratios ranging from 0.51 to 1.56. Last, accounting for all unaccounted sources of wild fish inputs, collateral fishing mortality, and adjusting for fed systems revealed FI:FO ratios between 0.57 and 1.78 (Fig. 2).

**Fig. 3.**
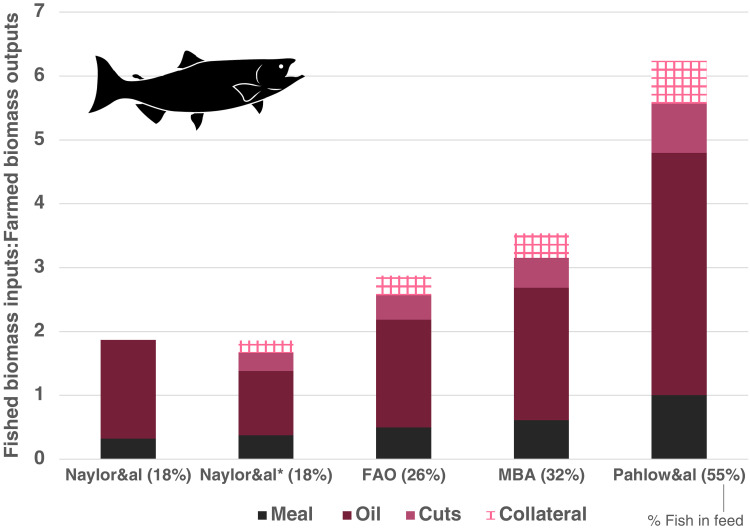
Biomass ratios of wild fish mortality to farmed fish product (FI:FO) for salmon farming, delineated by cause and repeated using four different datasets. The leftmost column represents results of the FI:FO formula outlined by RW Hardy and used in Naylor *et al.* (*14*). Fish inputs not captured by this method are included in Naylor&al* and subsequent scenarios. Cuts represent previously unaccounted wild fish classified as trimmings and by-products. Collateral represents mortality from bycatch and slipping during capture. Sources are Naylor *et al.* (*14*), Tacon *et al.* (*15*), Monterey Bay Aquarium Seafood Watch (*16*–*25*), and Pahlow *et al.* (*26*).

Examining the salmon species group, the highest-producing division of the fish farming industry using carnivorous feeds, oil use accounted for more than half of wild fish mortality in all scenarios, spanning FI:FO ratios from 1.86 to 6.24 when accounting for collateral mortality (Fig. 3). The adjusted reduction efficiency of fish oil used in the adjusted formula in the Naylor&al* scenario is favorable for salmon, but outweighed in scenarios using the other three sets of alternative parameters. Adjusting for unfed systems did not affect carnivorous species groups, as 100% of farmed salmon, trout, and eel are fed.

### Terrestrial-sourced feed

**Fig. 4.**
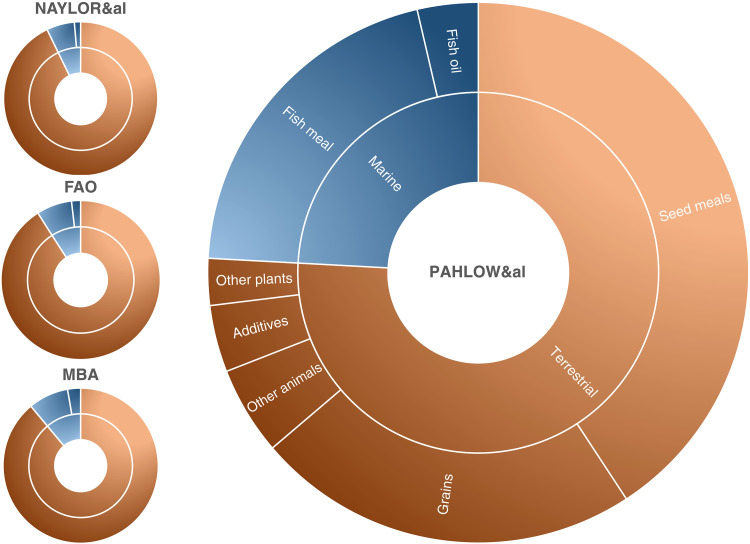
Aquaculture feed composition across four scenarios. Divisions within blue wedges represent proportions of fish meal and oil. Orange wedges without divisions represent datasets without information on the types and proportions of crops used. Sources are Naylor *et al.* (*14*), Tacon *et al.* (*15*), Monterey Bay Aquarium Seafood Watch (*16*–*25*), and Pahlow *et al.* (*26*).

Across the various feed composition scenarios, terrestrial-sourced ingredients comprised 76 to 93% of aquaculture feed. Detailed feed crop composition data were only available for Pahlow *et al.* (*26*), which we therefore used to estimate land and water use impacts (Fig. 4).

**Fig. 5.**
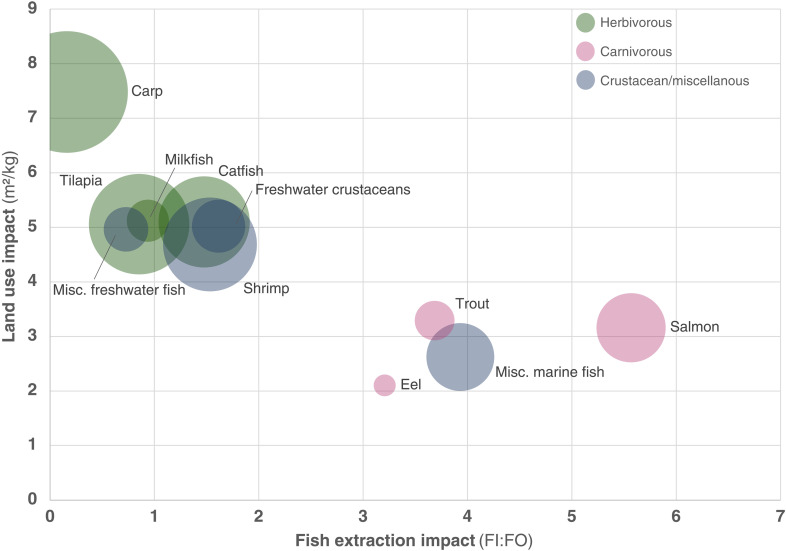
Fish extraction and land use trade-off in aquaculture. Circle sizes represent the total production weight of each farmed species group by reported production for the year 2015. Terrestrial impacts are calculated by multiplying feed use data from Pahlow *et al.* (*26*) by land use data from Poore and Nemecek (*44*). Fishing impacts are assessed as a ratio of fished biomass input over farmed fish biomass output (FI:FO), calculated using our adjusted FI:FO method and Pahlow *et al.* (*26*) feed composition data.

Comparing the environmental impacts of feed formulations across the aquaculture industry, species groups that are fed less wild fish generally have more intensive crop farming impacts. Species groups using herbivorous fish feeds consume more than twice as much land and 43% more fresh water as species groups that are fed carnivorous feeds (fig. S3). Despite high production from unfed systems, the most intensive aquaculture feed species group in terms of both land and water use was carp, due in part to the high use of rapeseed meal. Producing carnivorous feeds for groups such as salmon, trout, and eel uses less land and water in exchange for higher fish extraction impacts (Fig. 5).

**Fig. 6.**
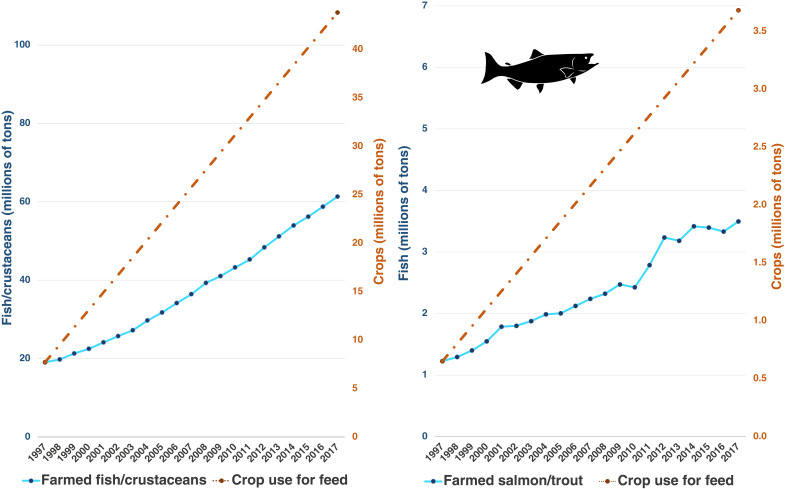
Crop use for aquaculture feed over time. Blue lines indicated reported production for all farmed fish and crustacean species groups combined in the left panel and for salmon and trout in the right panel. Orange trend lines indicate the increase in crop feed requirements calculated using 1997 and 2017 data on feed composition and FCR by species group from Naylor *et al.* (*14*).

Illustrating the ramifications of this trade-off, hypothetically adopting Naylor *et al.* (*14*) assumptions for improved FCRs and shifts from marine to terrestrial inputs between 1997 and 2017 requires a 468% aggregate increase in feed crops, while the reported biomass of farmed fish and crustaceans increased during this period by a factor of 222%. Put another way, the “crops-in:fish-out” ratio of the fish and crustacean aquaculture sector as a whole increased by 77%, from 0.40 to 0.71 (Fig. 6). This increase in crop use outpaced production gains in eight of nine major species groups, increasing at over twice the rate of production in tilapia, salmon, and eel and at more than three times the rate of production in milkfish. In only one group—catfish, in which the biomass of crop use already exceeded the total mass of production—did crop use intensity decrease over time (fig. S4). In some groups, such as salmonids, biomass of farmed fish and crustaceans outweighed crop use in the beginning of the time period and was overtaken by the end.

## DISCUSSION

The impact of aquaculture on wild fish is greater than commonly cited. Of the four feed composition datasets presented here, the parameters used by Naylor *et al.* (*14*) for the most referenced calculation of FI:FO (*14*) is also the dataset that demonstrates the lowest use of fish in global aquaculture feeds. In addition, if we take a broader and ecologically oriented approach to calculating the biomass of fish killed for aquaculture feed, a substantial fraction of the impact of aquaculture feed on marine food webs has been previously overlooked.

Figures used for feed composition data have extensive ramifications, as the FI:FO method of assessing fish use intensity is highly sensitive to errors, assumptions, and underreporting. Minor variations in parameters can have compounding effects on metrics. The accuracy of available data is difficult to evaluate; thus, the scenarios selected here incorporate the widest available range of peer-reviewed literature, offering a representative range of estimates. By replicating this exercise in scenarios, we aim to portray a less precise, yet therefore more accurate picture of the fish extraction impact of aquaculture.

Many additional forms of uncertainty still remain, and policy should reflect the risks of these still poorly characterized resource use requirements. Almost all parameters used in this study and others are self-reported by industry, taken or projected from voluntary surveys, as aquaculture feed corporations are generally not required to disclose feed formulations and often classify them as proprietary information.

Disclosure and inspection policies can both enhance transparency. Stable isotope analysis has begun to help disaggregate nutrient composition of feeds by using isotopic signatures to partition fractions of ingredients in aquaculture feed, such as crops, manure, and wild fish (*53*, *54*). Public and private policy interventions should recognize that opacity in feed formulation and use represents a barrier to the sustainability of the sector. If corporations voluntarily disclose and independently verify feed compositions, or are compelled to do so legally, the methods proposed by researchers could be evaluated and this opacity would be reduced.

Uncertainties in feed formulation are particularly consequential with regard to the use of fish oil. Less oil is extracted from fish than meal, which means variations in fish oil content are more consequential in calculating live weight equivalents of captured fish (*13*). Therefore, estimates for farmed species groups such as salmon that use oily carnivorous fish feed have higher variability and greater uncertainty.

In addition, the reduction efficiency of fish oil, in particular, can vary widely both between years and within years, because the body fat of small pelagic fishes fluctuates across species, seasons, and age demographics. Oil yield falls when, for instance, the anchoveta catch includes a high proportion of juveniles or has undergone an El Niño event. Since 2000, captured anchoveta have become smaller on average, with the highest size classes all but disappearing (*55*). It may be that the 7.6% fish oil reduction efficiency calculated for 2017 in this study and possibly even the 5% used by Naylor *et al.* (*14*) are overestimates going forward. While fluctuations caused by fish life cycles are difficult to capture, calculating reduction efficiencies from weighted averages of species in reported catch (data S1) can help quantify these variables more accurately than assigning constant values in future research.

Evaluating alternative parameters for feed composition, we find that a relatively small (17%) variation in proportion of marine-sourced feed more than tripled the final figures for aggregate efficiency of fish use. Pahlow *et al.* (*26*), the most fish-intensive feed scenario, is the only dataset obtained by metastudy, as opposed to surveying aquaculture feed manufacturers, although these data are still mainly from industry. These sources indicate higher proportions of fish inclusion than others, but are less up to date as a result of scarcer data. However, the MBA data are the most contemporary and yield the second highest FI:FO quotients. Last, the FAO dataset demonstrates how a small degree of underreporting might have compounding ramifications for the efficiency of wild fish use in fish and crustacean farming.

Comparing results calculated by Naylor *et al.* (*14*) to those derived from the same data using our adjusted calculations, conventional methodological assumptions tend to lower FI:FO ratios. While our calculations indicate that the efficiency of oil extraction from wild fish may be underestimated by Naylor *et al.* (*14*), attributed in part to the high reported use of sardines in the study year, the deduction of all trimmings does not accurately portray the reliance of aquaculture on wild capture. Industry disclosures suggest a full two-thirds of these trimmings are cut from wild fish, while by some definitions these cuts are classified as by-products, they are only one processing stage removed from wild catch and trimmings can comprise up to 70% of an animal’s biomass (*56*). Furthermore, as demand for aquaculture feed has risen, so has the economic value of trimmings, such that they may constitute a substantial proportion or even majority of a wild animal’s sale value, contributing to demand for their capture (*57*). Last, the “by-products” category includes millions of tons of whole fish, which are classified as trash due to being nontarget species. Therefore, we do not exclude wild fish trimmings from wild fish inputs.

Expanding further, collateral fishing mortality represents another substantial ecological impact of feed manufacturing that has gone largely unaccounted for due to the standard of limiting scope to fish inputs. Bycatch in fisheries not only includes other fish species, but also can include seabirds, turtles, marine mammals, and invertebrates. Moreover, the definition of bycatch fails to include slipping mortality because these fish are never hauled onto deck. While the convention is to quantify fish inputs, it may be more ecologically appropriate to consider fishing mortality. Therefore, we include, yet explicitly disaggregate, collateral mortality when evaluating the efficiency of fish and crustacean farming, as it is ultimately a question of the impact of the industry on wild fish populations.

Although the impacts of feed manufacturing are considerable, they represent a fraction of the environmental impacts of aquaculture as a whole. Unaccounted wild fish mortality extends far beyond feed, including abandoned gear (*58*, *59*), processing collateral (*60*), the capture of cleaner fish (*61*), and “ranched” species that cannot be bred in captivity, such as tuna and eel (*62*). Indirect mortality and broader environmental impacts also include transmission of pathogens (*63*), application of pesticides and antibiotics (*64*, *65*), outbreaks of introduced species (*66*, *67*), domestication of wild species (*68*), eutrophication (*69*, *70*), deoxygenation (*71*), chemical pollution (*72*, *73*), stream diversion (*74*), tidal disruption (*75*), coastal degradation (*76*), and greenhouse gas emissions—which may be underaccounted for (*77*) and are a priority for future research.

In terms of efficiency, the inclusion of unfed fish in the “fish out” denominator can give the impression that FI:FO estimates the efficiency of the fish feeding process, when it is actually an analysis of the industry at large, of which more than a quarter of production comes from unfed, primarily traditional, and commercially unavailable systems. Therefore, to provide estimates for average feeding efficiency, we have also provided calculations excluding fish farmed in unfed systems from FI:FO. As opposed to total industrial inputs over outputs, the aggregate FI:FO ratio of fish and crustacean feeding processes is more than a third higher: ranging from 0.51 to 1.56 before considering collateral mortality (Fig. 2).

Last, the impact of terrestrial feeds have been largely overlooked in discussions on aquaculture sustainability. Adopting the most widely cited scenario for efficiency gains in wild fish use requires that crop use would need to outpace the growth of aquaculture production. This illustrates that over time, aquaculture outputs do not decouple from inputs, but rather inputs shift from marine to terrestrial impacts.

While crop demand for aquaculture feed can be extrapolated across a time series, land and water use estimates are expressed here only as a snapshot, due to increases in crop yield efficiency over recent decades. However, the increase in the aquaculture sector’s total utilization of feed crops under the Naylor&al scenario—468% overall and 77% on a per-unit basis—has likely outpaced improvements in crop yields (tons of crops produced per hectare). Between 1997 and 2017, global maize and soy yields increased by 38 and 32%, respectively (*78*). This suggests that the land requirements demanded by aquaculture production have increased per unit of production and overall.

Considering marine and terrestrial inputs combined, these findings reiterate that fish and crustacean farming does not, on net, produce calories or protein (*79*). Retention of dietary nutrients in feed is less studied and more variable, but also a net loss (*80*). While some analyses have examined nutrient retention in a FI:FO framework, nutrient availability in terrestrial feed inputs must also be considered to make congruent comparisons with other food sectors. Future efforts should analyze the net micronutrient benefits and losses across various aquaculture species groups globally. Although aquaculture can provide concentrated sources of deficient nutrients in some contexts (*81*), it can diminish nutritional quality in others (*82*), and reduction fisheries remain a notable driver of malnutrition (*11*, *83*).

While FI:FO is informative in some contexts, it compares a small fraction of inputs to total outputs and omits the impacts of shifting to terrestrial feeds (*84*, *85*). The methods we provide do not equate to a full life cycle assessment (LCA), but provide a more accurate quantification of fish extraction and crop cultivation impacts, which is a prerequisite to more accurate LCAs in future research.

A wider set of criteria for sustainable policies and investments, beyond FI:FO, include numerous marine and terrestrial impacts of food production. Many alternative protein options have low terrestrial impacts and minimal marine impacts (*86*), but micronutrient differences remain a concern and nutritional profiles are in need of improvement. More broadly, this review widens the scope of sustainability considerations for aquaculture inputs, including bycatch, slipping mortality, and terrestrial impacts. However, it does not evaluate downstream environmental impacts, broader ecological impacts, or impacts on labor, public health, and animal welfare (*87*). Sustainable policies and investments into food production should be evaluated and compared across as many of these impacts as possible.

The expanded view of feeding global aquaculture offered here suggests that common sustainability accounting methods have been too narrow, overconfident in their precision, and overly optimistic. Both marine and terrestrial impacts are still highly uncertain, but these revised estimates suggest that the environmental impacts of this sector, in its current form and structure, are sufficiently large that directives to expand this sector on sustainability grounds should be reconsidered.

## Supplementary Materials

### The PDF file includes:

Figs. S1 to S4 Legend for data S1 References
