# Supplementary material for: Feeding global aquaculture
Source: Sci Adv. 2024 Oct 16;10(42):eadn9698. doi: 10.1126/sciadv.adn9698 (PMC11482329; doi:10.1126/sciadv.adn9698)
Supplement: Supplementary file 1 — Figs. S1 to S4 Legend for data S1 References [file sciadv.adn9698_sm.pdf]

Science Advances
AAAS

Supplementary Materials for

Feeding global aquaculture

Spencer Roberts *et al.*

Corresponding author: Matthew N. Hayek, matthew.hayek@nyu.edu

Sci. Adv. **10**, eadn9698 (2024)
DOI: 10.1126/sciadv.adn9698

The PDF file includes:

Figs. S1 to S4
Legend for data S1
References

Other Supplementary Material for this manuscript includes the following:

Data S1

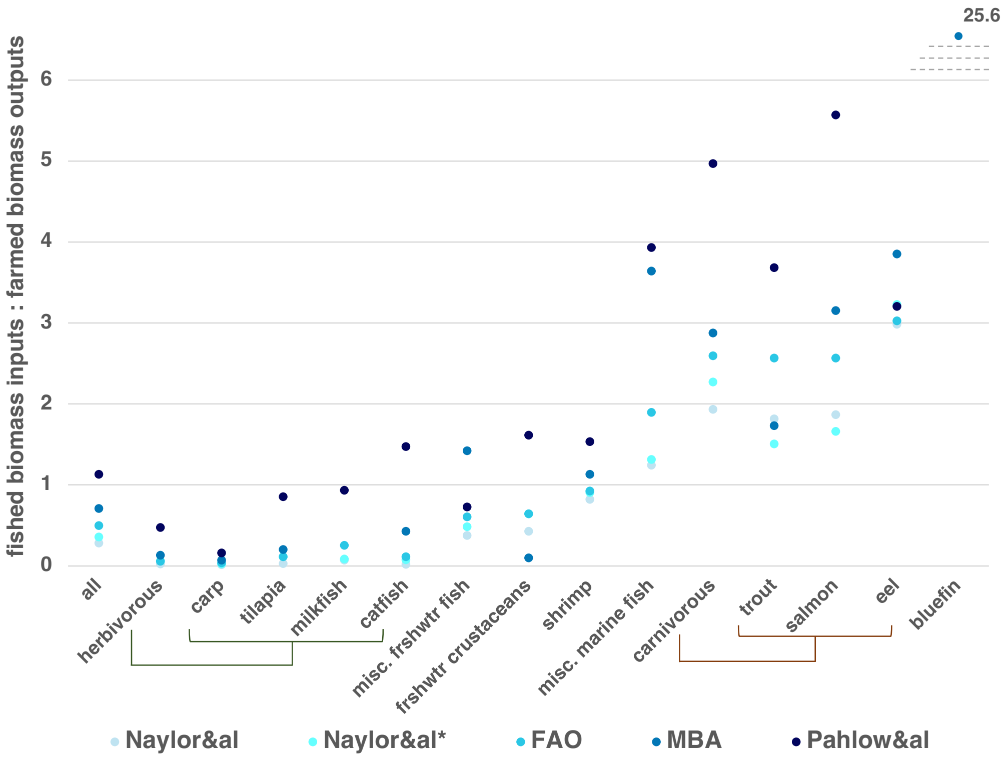

Figure S1. Calculated fished inputs : farmed outputs (FI:FO) biomass ratios by aquaculture species group using two different methods and four different feed composition scenarios, a visualization of Table 1. The leftmost column represents results of the FI:FO formula outlined by RW Hardy and used in Naylor et al. (14) 2021. Fish inputs not captured by this method are included in Naylor* and subsequent scenarios. Collateral fishing mortality is not added and unfed systems are not subtracted here. Data sets differ in terms of efficiency rates of fish meal and oil processing, meal and oil inclusion in feed, and for MBA, percent of each from byproducts. Figures are selected as closely to 2017 as available. Feed composition sources are Naylor et al. (14), Tacon et al. (15), Monterey Bay Aquarium Seafood Watch (16–25), and Pahlow et al. (26).

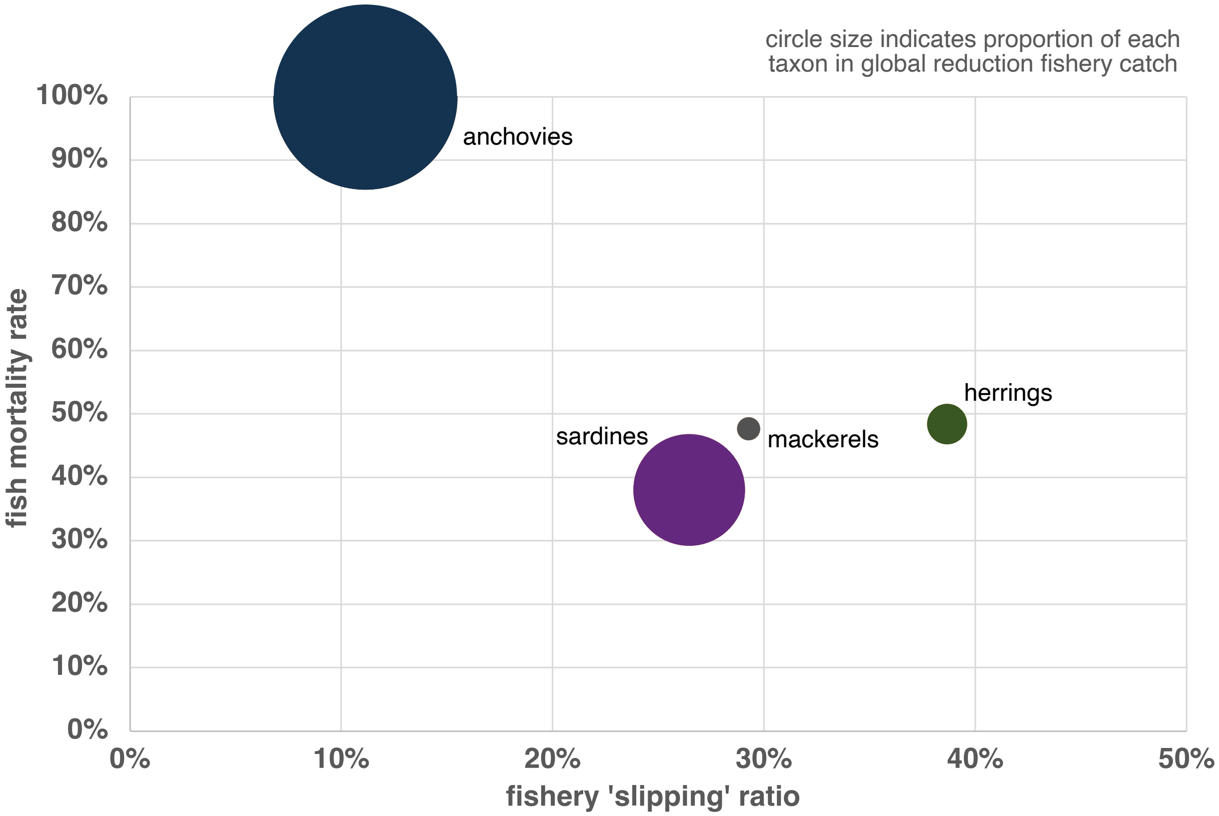

Figure S2. Unaccounted mortality in reduction fisheries. Slipping ratios and mortality rates for each taxon represent averages of values reviewed in literature, referenced in Table S3. Circle sizes represent proportions of each taxon in global reduction fishery catch, calculated in Table S3 using data from Green for SEAFISH (27).

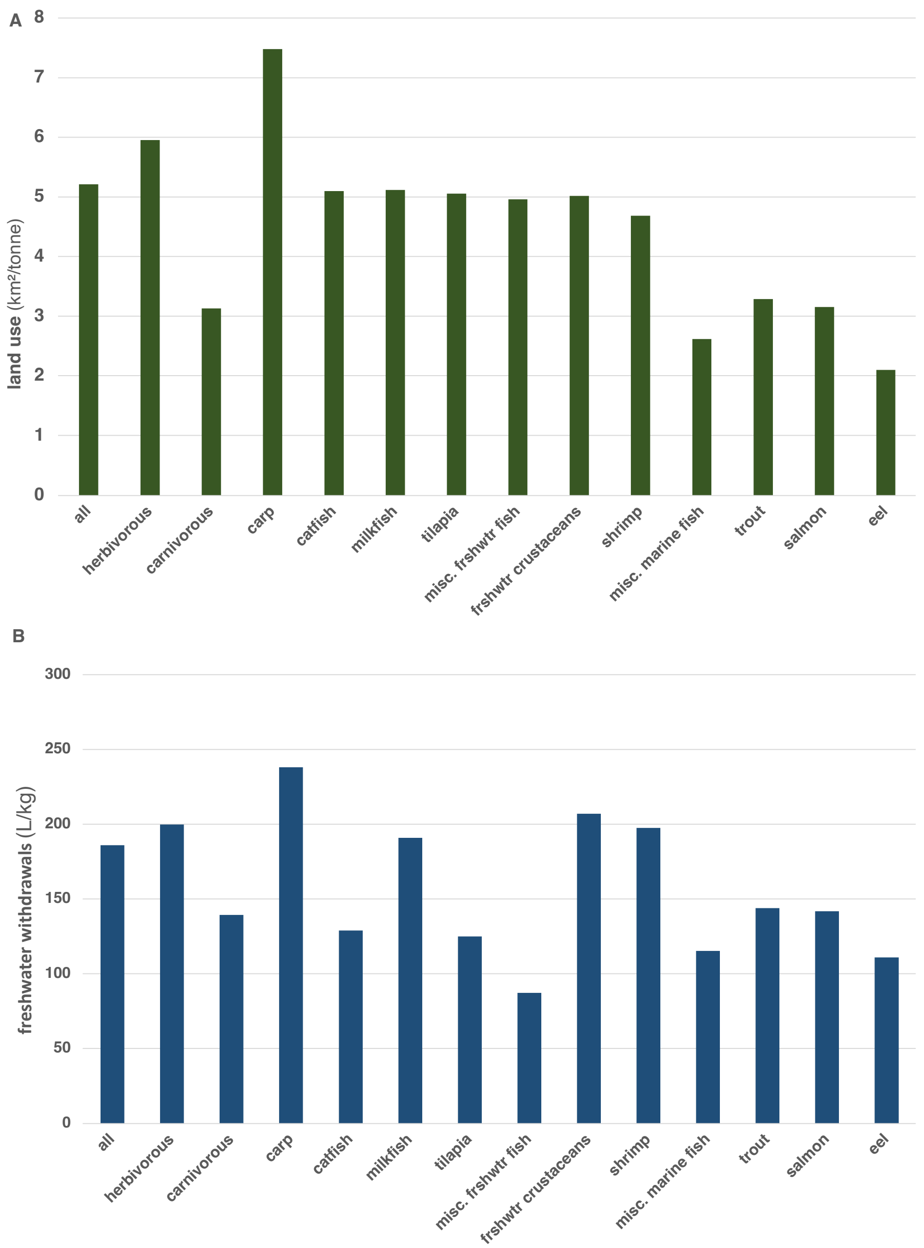

Figure S3. Land and water use in feed manufacturing by aquaculture species group. A. Land use is calculated by binning and weighting proportions of feed crop composition for various farmed species compiled by Pahlow et al. (26) into species groups and multiplying the proportion of each crop by land use per kilogram calculated by Poore & Nemecek (44). B. Freshwater withdrawals are calculated by binning and weighting "blue water" use for various farmed species compiled by Pahlow et al. (26) into species groups.

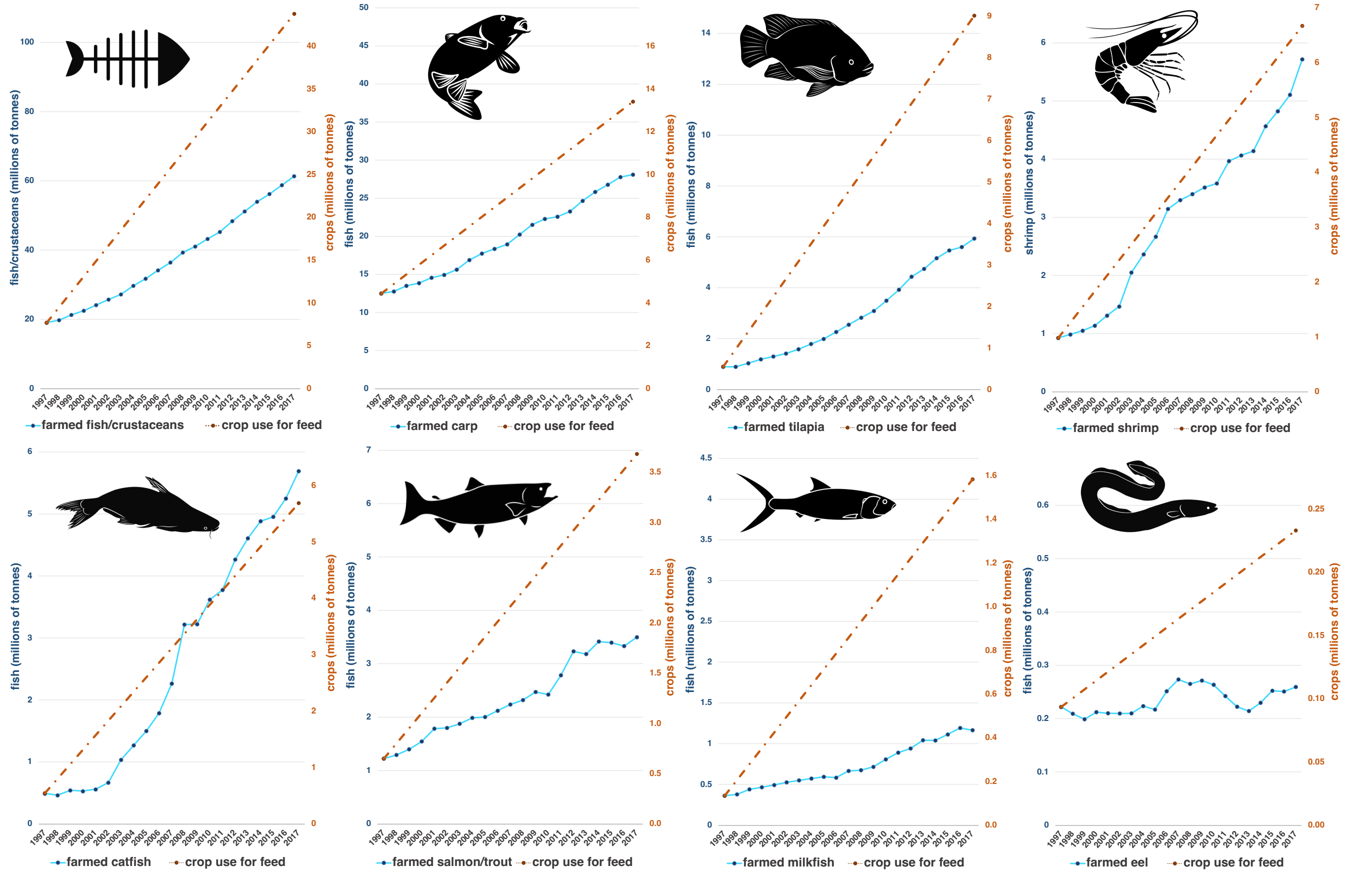

Figure S4. Crop use over time by aquaculture species group under decreasing intensity of wild fish use calculated by Naylor et al. 2021 (14). Total crop consumption by species group is calculated by multiplying reported fed fish production by feed conversion ratio and proportion of terrestrially-sourced ingredients. Initial values are taken from Naylor et al. 2000 (52). Final values are taken from Naylor et al. 2021 (14). Annual reported farmed fish by species group and for all fish and crustacea are taken from FAO FishStatJ (88).

Data S1. All calculations used in the analysis, including the following: (worksheet 1) fish biomass on : fish biomass out ratios, including Table S1; (worksheet 2) terrestrial feed requirements and environmental impacts; (worksheet 3) crop use over time; (worksheet 4) Reduction Efficiencies of Meal and Oil from Whole Fish and Trimmings, including Table S2; (worksheet 5) unaccounted mortality in reduction fisheries; (worksheet 6) FAO aquaculture market data for production by species subgroup; (worksheet 7) environmental impacts of terrestrial feeds.

REFERENCES AND NOTES

1. J. Alder, B. Campbell, V. Karpouzi, K. Kaschner, D. Pauly, Forage fish: From ecosystems to markets. *Annu. Rev. Env. Resour.* **33**, 153–166 (2008).

2. A. G. J. Tacon, M. Metian, Fishing for aquaculture: Non-food use of small pelagic forage fish—A global perspective. *Rev. Fish. Sci.* **17**, 305–317 (2009).

3. T. Cashion, The end use of marine fisheries landings. *Fish. Cent. Res. Rep.* **24**, 108 (2016).

4. P. K. Sarker, Microorganisms in fish feeds, technological innovations, and key strategies for sustainable aquaculture. *Microorganisms* **11**, 439 (2023).

5. T. Cashion, P. Tyedmers, R. W. R. Parker, Global reduction fisheries and their products in the context of sustainable limits. *Fish Fish.* **18**, 1026–1037 (2017).

6. R. C. Urbina, L. L. Cruz, R. Castillo, Distribución y biomasa de anchoveta (*Engraulis ringens*) y múnida (*Pleuroncodes monodon*) en el ecosistema marino de la Reserva Nacional de Paracas, región sur del Perú: Distribution and biomass of anchovy (*Engraulis ringens*) and pelagic red squat lobster (*Pleuroncodes monodon*) in marine ecosystem of Paracas National Reserve, southern Peru. *Bol. Investig. Mar. Costeras* **51**, 99–116 (2022).

7. P. M. Cury, I. L. Boyd, S. Bonhommeau, T. Anker-Nilssen, R. J. M. Crawford, R. W. Furness, J. A. Mills, E. J. Murphy, H. Österblom, M. Paleczny, J. F. Piatt, J.-P. Roux, L. Shannon, W. J. Sydeman, Global seabird response to forage fish depletion—One-third for the birds. *Science* **334**, 1703–1706 (2011).

8. P. Majluf, S. De la Puente, V. Christensen, The little fish that can feed the world. *Fish Fish.* **18**, 772–777 (2017).

9. T. Cashion, F. Le Manach, D. Zeller, D. Pauly, Most fish destined for fishmeal production are food-grade fish. *Fish Fish.* **18**, 837–844 (2017).

10. W. A. Dunaway, M. C. Macabuac, The Asian fishery crisis, nutritional unequal exchange and food insecurity, in *Where Shrimp Eat Better than People*, W. A. Dunaway, M. C. Macabuac, Eds. (Brill, 2022), p. 31–89.

11. K. L. Nash, M. A. MacNeil, J. L. Blanchard, P. J. Cohen, A. K. Farmery, N. A. J. Graham, A. L. Thorne-Lyman, R. A. Watson, C. C. Hicks, Trade and foreign fishing mediate global marine nutrient supply. *Proc. Natl. Acad. Sci.* **119**, e2120817119 (2022).

12. A. G. J. Tacon, M. Metian, Global overview on the use of fish meal and fish oil in industrially compounded aquafeeds: Trends and future prospects. *Aquaculture* **285**, 146–158 (2008).

13. R. L. Naylor, R. W. Hardy, D. P. Bureau, A. Chiu, M. Elliott, A. P. Farrell, I. Forster, D. M. Gatlin, R. J. Goldburg, K. Hua, P. D. Nichols, Feeding aquaculture in an era of finite resources. *Proc. Natl. Acad. Sci.* **106**, 15103–15110 (2009).

14. R. L. Naylor, R. W. Hardy, A. H. Buschmann, S. R. Bush, L. Cao, D. H. Klinger, D. C. Little, J. Lubchenco, S. E. Shumway, M. Troell, A 20-year retrospective review of global aquaculture. *Nature* **591**, 551–563 (2021).

15. A. G. J. Tacon, M. R. Hasan, M. Metian, *Demand and Supply of Feed Ingredients for Farmed Fish and Crustaceans* (Food and Agriculture Organization of the United Nations, 2011).

16. Seafood Watch Consulting Researchers, Tilapia (2018). https://seafoodwatch.org/globalassets/sfw-data-blocks/reports/T/MBA_SeafoodWatch_TilapiaChinaReport.pdf.

17. Seafood Watch Consulting Researchers, Giant Tiger Prawn and White Shrimp (2021). https://seafoodwatch.org/globalassets/sfw-data-blocks/reports/S/MBA_SeafoodWatch_Shrimp_Malaysia.pdf.

18. Seafood Watch Consulting Researchers, Channel Catfish (2017). https://seafoodwatch.org/globalassets/sfw-data-blocks/reports/C/MBA_SeafoodWatch_ChannelCatfish_ChinaPonds_Report.pdf.

19. Seafood Watch Consulting Researchers, Gilthead Seabream, European Seabass and Meagre (2020). https://seafoodwatch.org/globalassets/sfw-data-blocks/reports/s/mba_seafoodwatch_seabream_seabass_meagre_report.pdf.

20. Aquaculture Stewardship Council, Aquaculture Stewardship Council Salmon Standard (2017). https://seafoodwatch.org/globalassets/sfw/pdf/eco-certification-benchmarks/seafood-watch-eco-certification-benchmark-asc-farmed-salmon.pdf.

21. J. H. Tidwell, Freshwater Prawns (2014). https://seachoice.org/wp-content/uploads/2015/04/MBA_SeafoodWatch_Freshwater_Prawn_Asia_Report.pdf.

22. Seafood Watch Staff, Barramundi (2021). https://seafoodwatch.org/globalassets/sfw-data-blocks/reports/b/mba_seafoodwatch_perch_giant_barramundi_vietnam_report.pdf.

23. Seafood Watch Consulting Researchers, Rainbow Trout (2023). https://seafoodwatch.org/globalassets/sfw-data-blocks/reports/t/seafood-watch-rainbow-trout-chile-28292.pdf.

24. J. Stoner, G. Banner-Stevens, Freshwater Eels (2014). https://seafoodwatch.org/globalassets/sfw-data-blocks/reports/E/MBA_SeafoodWatch_Eel_Asia.pdf.

25. C. Ma, Pacific Bluefin Tuna (2021). https://seafoodwatch.org/globalassets/sfw-data-blocks/reports/T/MBA_SeafoodWatch_Farmed_bluefin_tuna_Japan.pdf.

26. M. Pahlow, P. R. van Oel, M. M. Mekonnen, A. Y. Hoekstra, Increasing pressure on freshwater resources due to terrestrial feed ingredients for aquaculture production. *Sci. Total Environ.* **536**, 847–857 (2015).

27. K. Green, Fishmeal and fish oil facts and figures. Seafish (2016). https://seafish.org/document/?id=30fa924f-6f82-451d-90d7-60ba59c8c4bc.

28. P. Edwards, H. Anh Tuan, G. Allan, A survey of marine trash fish and fish meal as aquaculture feed ingredients in Vietnam. *Aust. Cent. Int. Agric. Res. Work. Pap.* **57**, 118373 (2004).

29. M. Aditi, L. Varsha, Fish processing: Product and by-product, processing and marketing. *Int. J. Fish. Aquat. Stud.*, **8**, 74–77. (2020).

30. Y. Stratoudakis, A. Marçalo, Sardine slipping during purse-seining off northern Portugal. *ICES J. Mar. Sci.* **59**, 1256–1262 (2002).

31. M. Tenningen, F. Zimmermann, K. Enberg, Pre-catch and discard mortality in Northeast Atlantic herring and mackerel fisheries: Consequences for stock estimates and advice. *ICES J. Mar. Sci.* **78**, 2603–2614 (2021).

32. R. Tejerina, M. Hermida, G. Faria, J. Delgado, The purse-seine fishery for small pelagic fishes off the Madeira Archipelago. *Afr. J. Mar. Sci.* **41**, 373–383 (2019).

33. L. Fauconnet, C. K. Pham, A. Canha, P. Afonso, H. Diogo, M. Machete, H. M. Silva, F. Vandeperre, T. Morato, An overview of fisheries discards in the Azores. *Fish. Res.* **209**, 230–241 (2019).

34. T. C. Borges, K. Erzini, L. Bentes, M. E. Costa, J. M. S. Gonçalves, P. G. Lino, C. Pais, J. Ribeiro, By-catch and discarding practices in five Algarve (southern Portugal) métiers. *J. Appl. Ichthyol.* **17**, 104–114 (2001).

35. J. Ruiz, M. Louzao, I. Oyarzabal, L. Arregi, E. Mugerza, A. Uriarte, The Spanish purse-seine fishery targeting small pelagic species in the Bay of Biscay: Landings, discards and interactions with protected species. *Fish. Res.* **239**, 105951 (2021).

36. D. A. Ramírez, *Alternativas tecnológicas para el control de descartes y reduccion de captura de juveniles en la pesqueria de anchoveta* (Oceana, 2018).

37. R. E. Olsen, F. Oppedal, M. Tenningen, A. Vold, Physiological response and mortality caused by scale loss in Atlantic herring. *Fish. Res.* **129–130**, 21–27 (2012).

38. M. Tenningen, A. Vold, R. E. Olsen, The response of herring to high crowding densities in purse-seines: Survival and stress reaction. *ICES J. Mar. Sci.* **69**, 1523–1531 (2012).

39. I. Huse, A. Vold, Mortality of mackerel (*Scomber scombrus L.*) after pursing and slipping from a purse seine. *Fish. Res.* **106**, 54–59 (2010).

40. S. J. Lockwood, M. G. Pawson, D. R. Eaton, The effects of crowding on mackerel (*Scomber scombrus* L.)—Physical condition and mortality. *Fish. Res.* **2**, 129–147 (1983).

41. R. W. Mitchell, S. J. Blight, D. J. Gaughan, I. W. Wright, Does the mortality of released Sardinops sagax increase if rolled over the headline of a purse seine net? *Fish. Res.* **57**, 279–285 (2002).

42. F. O. Düzbastılar, Z. Tosunoğlu, T. Ceyhan, M. H. Kaykaç, C. Aydın, Ö. Güleç, G. Metin, A variation in the mortality of european anchovy and european pilchard after sieving and discarding from a purse seine fishery in the Eastern Mediterranean. *Turk. J. Fish. Aquat. Sci.* **23**, 21516 (2022).

43. W. Malcorps, B. Kok, M. van't Land, M. Fritz, D. van Doren, K. Servin, P. van der Heijden, R. Palmer, N. A. Auchterlonie, M. Rietkerk, M. J. Santos, S. J. Davies, The sustainability conundrum of fishmeal substitution by plant ingredients in shrimp feeds. *Sustainability* **11**, 1212 (2019).

44. J. Poore, T. Nemecek, Reducing food's environmental impacts through producers and consumers. *Science* **360**, 987–992 (2018).

45. M. N. Hayek, H. Harwatt, W. J. Ripple, N. D. Mueller, The carbon opportunity cost of animal-sourced food production on land. *Nat. Sustain.* **4**, 21–24 (2021).

46. R. W. Newton, S. Maiolo, W. Malcorps, D. C. Little, Life cycle inventories of marine ingredients. *Aquaculture* **565**, 739096 (2023).

47. A. G. J. Tacon, Trends in global aquaculture and aquafeed production: 2000–2017. *Rev. Fish. Sci. Aquac.* **28**, 43–56 (2020).

48. National Research Council, *Nutrient Requirements of Fish and Shrimp* (The National Academies Press, 2011).

49. T. Ytrestøyl, T. S. Aas, T. Åsgård, Utilisation of feed resources in production of Atlantic salmon (*Salmo salar*) in Norway. *Aquaculture* **448**, 365–374 (2015).

50. K. Kelleher, *Discards in the World's Marine Fisheries. An Update*. (Food and Agriculture Organization of the United Nations, 2005).

51. D. Zeller, T. Cashion, M. Palomares, D. Pauly, Global marine fisheries discards: A synthesis of reconstructed data. *Fish Fish.* **19**, 30–39 (2018).

52. R. L. Naylor, R. J. Goldburg, J. H. Primavera, N. Kautsky, M. C. M. Beveridge, J. Clay, C. Folke, J. Lubchenco, H. Mooney, M. Troell, Effect of aquaculture on world fish supplies. *Nature* **405**, 1017–1024 (2000).

53. J. Gamboa-Delgado, Isotopic techniques in aquaculture nutrition: State of the art and future perspectives. *Rev. Aquac.* **14**, 456–476 (2021)

54. J. Narimbi, D. Mazumder, J. Sammut, Stable isotope analysis to quantify contributions of supplementary feed in Nile Tilapia *Oreochromis niloticus* (GIFT strain) aquaculture. *Aquacult. Res.* **49**, 1866–1874 (2018).

55. R. R. Reynaga Rojas, *Impacto económico de la sobrepesca por crecimiento en la pesca industrial de la anchoveta (Engraulis ringens), durante el periodo 2000–2019* (Universidad Nacional Agraria La Molina, 2023).

56. A.-L. Välimaa, S. Mäkinen, P. Mattila, P. Marnila, A. Pihlanto, M. Mäki, J. Hiidenhovi, Fish and fish side streams are valuable sources of high-value components. *Food Qual. Saf.* **3**, 209–226 (2019).

57. D. Coppola, C. Lauritano, F. Palma Esposito, G. Riccio, C. Rizzo, D. de Pascale, Fish waste: From problem to valuable resource. *Mar. Drugs* **19**, 116 (2021).

58. D. Laist, Marine debris entanglement and ghost fishing: A cryptic and significant type of bycatch?, in *Solving Bycatch: Considerations for Today and Tomorrow* (National Agrarian University, 1995).

59. G. Macfadyen, T. Huntington, R. Cappell, *Abandoned, Lost or Otherwise Discarded Fishing Gear* (Food and Agriculture Organization of the United Nations, 2009).

60. B.C. salmon farms linked to explosive spike in wild fish deaths, *Coast Reporter* (2023). https://coastreporter.net/highlights/bc-salmon-farms-linked-to-explosive-spike-in-wild-fish-deaths-7915118.

61. E. Faust, E. Jansson, C. André, K. T. Halvorsen, G. Dahle, H. Knutsen, M. Quintela, K. A. Glover, Not that clean: Aquaculture-mediated translocation of cleaner fish has led to hybridization on the northern edge of the species' range. *Evol. Appl.* **14**, 1572–1587 (2021).

62. A. Lovatelli, P. F. Holthus, Food and Agriculture Organization of the United Nations, Eds. *Capture-Based Aquaculture: Global Overview* (Food and Agriculture Organization of the United Nations, 2008).

63. A. G. Murray, Epidemiology of the spread of viral diseases under aquaculture. *Curr. Opin. Virol.* **3**, 74–78 (2013).

64. S. Ray, S. T. Shaju, Bioaccumulation of pesticides in fish resulting toxicities in humans through food chain and forensic aspects. *Environ. Anal. Health Toxicol.* **38**, e2023017 (2023).

65. E. Kramarsky-Winter, C. A. Downs, A. Downs, Y. Loya, Cellular responses in the coral *Stylophora pistillata* exposed to eutrophication from fish mariculture. *Evol. Ecol. Res.* **11**, 381–401 (2009).

66. T. L. F. Leung, A. E. Bates, More rapid and severe disease outbreaks for aquaculture at the tropics: Implications for food security. *J. Appl. Ecol.* **50**, 215–222 (2013).

67. L. E. Escobar, S. Mallez, M. McCartney, C. Lee, D. P. Zielinski, R. Ghosal, P. G. Bajer, C. Wagner, B. Nash, M. Tomamichel, P. Venturelli, P. P. Mathai, A. Kokotovich, J. Escobar-Dodero, N. B. D. Phelps, Aquatic invasive species in the great lakes region: An overview. *Rev. Fish. Sci. Aquac.* **26**, 121–138 (2018).

68. C. M. Duarte, N. Marbá, M. Holmer, Rapid domestication of marine species. *Science* **316**, 382–383 (2007).

69. R. J. Gowen, Managing eutrophication associated with aquaculture development. *J. Appl. Ichthyol.* **10**, 242–257 (1994).

70. H. Herliwati, M. Rahman, Loading capacity of water pollution from cage aquaculture in south kalimantan rivers. *J. Wetl. Environ. Manag.* **10**, 1–11 (2022).

71. X. Zhang, C. Yao, B. Zhang, W. Tan, J. Gong, G. Wang, J. Zhao, X. Lin, Dynamics of benthic nitrate reduction pathways and associated microbial communities responding to the development of seasonal deoxygenation in a coastal mariculture zone. *Environ. Sci. Technol.* **57**, 15014–15025 (2023).

72. D. P. Weston, Ecological effects of the use of chemicals in aquaculture (2000); http://hdl.handle.net/1834/40241.

73. J. V. Tarazona, M. J. Muñoz, G. Carbonell, M. Carballo, J. A. Ortiz, A. Castaño, A toxicological assessment of water pollution and its relationship to aquaculture development in Algeciras Bay, Cadiz Spain, *Arch. Environ. Contam. Toxicol.* **20**, 480–487 (1991).

74. F. O'Donncha, M. Hartnett, S. Nash, Physical and numerical investigation of the hydrodynamic implications of aquaculture farms. *Aquac. Eng.* **52**, 14–26 (2013).

75. F. O. Donohue, *Physical and Numerical Modelling of Impeded Tidal Flows: Effects of Aquaculture Structures on Hydrodynamics and Material Transport* (University of Galway, 2012).

76. B. K. van Wesenbeeck, T. Balke, P. van Eijk, F. Tonneijck, H. Y. Siry, M. E. Rudianto, J. C. Winterwerp, Aquaculture induced erosion of tropical coastlines throws coastal communities back into poverty. *Ocean Coast. Manag.* **116**, 466–469 (2015).

77. M. F. Astudillo, G. Thalwitz, F. Vollrath, Modern analysis of an ancient integrated farming arrangement: Life cycle assessment of a mulberry dyke and pond system. *Int. J. Life Cycle Assess.* **20**, 1387–1398 (2015).

78. UN FAO, FAOSTAT database (2024). http://faostat.fao.org/site/368/default.aspx#ancor.

79. J. P. Fry, N. A. Mailloux, D. C. Love, M. C. Milli, L. Cao, Feed conversion efficiency in aquaculture: Do we measure it correctly? *Environ. Res. Lett.* **13**, 024017 (2018).

80. D. F. Willer, R. Newton, W. Malcorps, B. Kok, D. Little, A. Lofstedt, B. de Roos, J. P. W. Robinson, Wild fish consumption can balance nutrient retention in farmed fish. *Nat. Food* **5**, 221–229 (2024).

81. Q. Xu, L. Dai, P. Gao, Z. Dou, The environmental, nutritional, and economic benefits of rice-aquaculture animal coculture in China. *Energy* **249**, 123723 (2022).

82. J. R. Bogard, S. Farook, G. C. Marks, J. Waid, B. Belton, M. Ali, K. Toufique, A. Mamun, S. H. Thilsted, Higher fish but lower micronutrient intakes: Temporal changes in fish consumption from capture fisheries and aquaculture in Bangladesh. *PLOS ONE* **12**, e0175098 (2017).

83. C. C. Hicks, P. J. Cohen, N. A. J. Graham, K. L. Nash, E. H. Allison, C. D'Lima, D. J. Mills, M. Roscher, S. H. Thilsted, A. L. Thorne-Lyman, M. A. MacNeil, Harnessing global fisheries to tackle micronutrient deficiencies. *Nature* **574**, 95–98 (2019).

84. AquaFeed, Low-cost aquafeed reduces operational costs in fish farms (2021). https://aquafeed.com/newsroom/news/low-cost-aquafeed-reduces-operational-costs-in-fish-farms/.

85. B. Zlaugotne, J. Pubule, D. Blumberga, Advantages and disadvantages of using more sustainable ingredients in fish feed. *Heliyon* **8**, e10527 (2022).

86. R. E. Santo, B. F. Kim, S. E. Goldman, J. Dutkiewicz, E. M. B. Biehl, M. W. Bloem, R. A. Neff, K. E. Nachman, Considering plant-based meat substitutes and cell-based meats: A public health and food systems perspective. *Front. Sustain. Food Syst.* **4**, 134 (2020).

87. B. Franks, C. Ewell, J. Jacquet, Animal welfare risks of global aquaculture. *Sci. Adv.* **7**, eabg0677 (2021).

88. FAO, FAO fisheries and aquaculture division (2023). www.fao.org/fishery/en/statistics/software/fishstatj.
